# Photoresponsive nanoclusters open an avenue for nanofabrication

**DOI:** 10.1093/nsr/nwag136

**Published:** 2026-03-05

**Authors:** Avirup Sardar, Rongchao Jin

**Affiliations:** Department of Chemistry, Carnegie Mellon University, USA; Department of Chemistry, Carnegie Mellon University, USA

At a time of skyrocketing demands for fast data storage and computation power, photoresponsive materials could be the next big thing owing to their remarkable controllability by using optical, chemical and morphological handles. For quite some time, the photoresponsiveness of small molecules (especially in the solution state) has been studied. However, for real-world applications, obtaining efficient photoresponse in the solid state remains a challenge [1–3].

Lately, a new class of nanomaterials—atomically precise nanoclusters (NCs) of metals and alloys—have emerged, which act as a bridge between small molecules and regular nanoparticles by exhibiting discrete electronic states and well-defined structures. The optical properties of NCs can be tuned by varying the number of atoms, ligands, heterometallic doping or changing their shape [4,5], which makes the NCs potentially promising for atomic-level photoinduced transformations.

In a recent study [6], Zhu and co-workers achieved an efficient and controllable photoinduced structural transformation in the solid state by using homometallic copper (Cu) and silver (Ag)-doped copper NCs. Although the photoresponsivity of materials has been previously reported in the solution phase, transformation in the solid state, and, moreover, achieving a high level of controllability, is still limited. Compared with solution systems, the solid state poses several barriers, including molecular rigidity, fixed spatial arrangements and tight packing, which all effectively overwhelm the photoactivity and thus raise an open challenge—how to engineer molecular materials for a quick, efficient and controllable photoresponse that is not restricted by the limitations of the solid state.

In their work [6], the authors synthesized and crystallized two NCs: Cu_18_ and single-Ag-atom-doped AgCu_17_, both of which transform into Cu_14_ under 365 nm of ultraviolet (UV)

irradiation. Interestingly, the Ag-doped counterpart shows a 3-fold enhancement in the conversion efficiency for the formation of Cu_14_ when compared with the undoped Cu_18_. Such an efficiency boost is attributed to the intricate changes in the electronic structure introduced by substitution of the innermost Cu atom with Ag. Specifically, doping leads to a direct kinetic advantage for AgCu_17_, in which the Ag atom promotes faster transformation into the metastable intermediate that produces a greater accumulation of the target Cu_14_ before eventual degradation. Also, the irradiation time plays an important role, as shortening the time leads to no transformation while a longer time leads to the destruction of the NCs. Mechanistically, the transformation comprises electronic excitation, ligand dynamics and core restructuring, emphasizing the multilevel nature of NC photochemistry.

**Figure 1.**
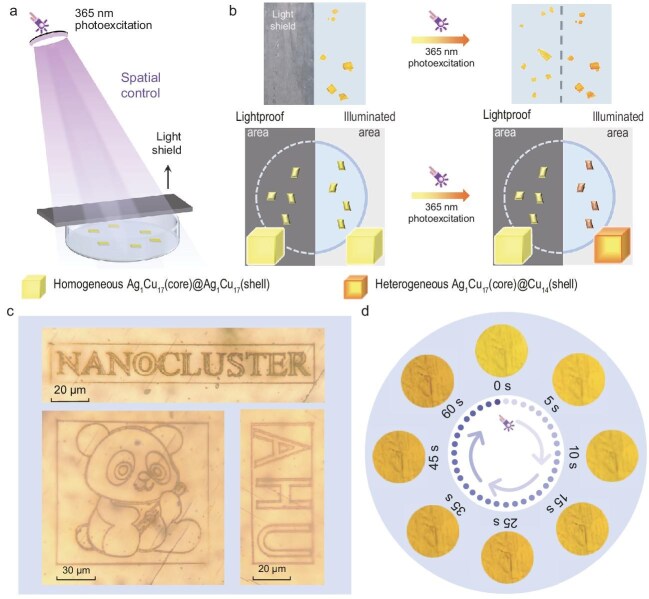
Spatial and temporal control over photoinduced conversion from AgCu_17_ to Cu_14_ NCs. (a) Device diagram. (b) Color changes before and after 365-nm photoexcitation in lightproof or illuminated areas of the NCs. Insets: illustrations of simulated NC-assembled crystals (homogeneous or heterogeneous). (c) Nanopatterning on the surface of a crystal. (d) Color evolution in a crystal upon UV irradiation. Reproduced from [6] with permission.

Most interestingly, the authors were able to precisely control the photoresponse at specific parts of the NC-assembled crystal. Under UV illumination, only the areas under the laser spot were subject to photoinduced transformation, while the region under a photo shield had no change at all. Further, the authors demonstrated micro-nanometer-scale spatial control by using femtosecond cold laser technology. As shown in Fig. 1, amazingly, letters and various patterns can be inscribed in the crystals, which are found to have nanoscale dimensions just by photopatterning the illuminated area from AgCu_17_ to Cu_14_. Diving deeper into the process, through UV–vis and mass spectrometry, the authors propose that the NCs only transformed in the outer layers of the crystal, while the inner layers remain intact, hence forming a Cu_14_/AgCu_17_ core–shell arrangement. Thus, the color-changed (irradiated) NCs are heterogeneous and the color-maintained crystals (not irradiated) are homogeneous.

In summary, this work [6] shows that NCs can be a promising material for nanofabrication and nanodevice applications through efficient, controllable and programmable photoinduced transformations. By introducing the effects of doping into structural transformation, this study establishes a general framework for designing future photoresponsive materials in the solid state, opening a new avenue in nanoengineering.
